# A novel faculty development tool for writing a letter of recommendation

**DOI:** 10.1371/journal.pone.0244016

**Published:** 2020-12-16

**Authors:** Kris Saudek, Robert Treat, Amanda Rogers, Danita Hahn, Sara Lauck, David Saudek, Michael Weisgerber

**Affiliations:** 1 Division of Neonatology, Department of Pediatrics, Medical College of Wisconsin, Milwaukee, Wisconsin, United States of America; 2 Department of Academic Affairs, Medical College of Wisconsin, Milwaukee, Wisconsin, United States of America; 3 Department of Pediatrics, Division of Hospital Medicine, Milwaukee, Wisconsin, United States of America; 4 Department of Pediatrics, Division of Cardiology, Milwaukee, Wisconsin, United States of America; Lundquist Institute at Harbor-UCLA Medical Center, UNITED STATES

## Abstract

**Objective:**

Based on a national survey of program directors we developed a letter of recommendation (LOR) scoring rubric (SR) to assess LORs submitted to a pediatric residency program. The objective was to use the SR to analyze: the consistency of LOR ratings across raters and LOR components that contributed to impression of the LOR and candidate.

**Methods:**

We graded 30 LORs submitted to a pediatric residency program that were evenly distributed based on final rank by our program. The SR contained 3 sections (letter features, phrases, and applicant abilities) and 2 questions about the quality of the LOR (LORQ) and impression of the candidate (IC) after reading the LOR on a 5-point Likert scale. Inter-rater reliability was calculated with intraclass correlation coefficients (ICC(2,1)). Pearson (r) correlations and stepwise multivariate linear regression modeling predicted LORQ and IC. Mean scores of phrases, features, and applicant abilities were analyzed with ANOVA and Bonferroni correction.

**Results:**

Phrases (ICC(2,1) = 0.82, p<0.001)) and features (ICC(2,1) = 0.60, p<0.001)) were rated consistently, while applicant abilities were not (ICC(2,1) = 0.28, p<0.001)). For features, LORQ (R^2^ = 0.75, p<0.001) and IC (R^2^ = 0.58, p<0.001) were best predicated by: writing about candidates’ abilities, strength of recommendation, and depth of interaction with the applicant. For abilities, LORQ (R^2^ = 0.47, p<0.001) and IC (R^2^ = 0.51, p<0.001) were best predicted by: clinical reasoning, leadership, and communication skills (0.2). There were significant differences for phrases and features (p<0.05).

**Conclusions:**

The SR was consistent across raters and correlates with impression of LORQ and IC. This rubric has potential as a faculty development tool for writing LORS.

## Introduction

The letter of recommendation (LOR) is valued by program directors (PDs) when making decisions about candidates to interview and rank in their programs, yet little is known about how these documents sway PDs and contribute to those decisions [1–3]. While some have suggested LORs do shed insight into who is likely to perform well during residency, other literature does not support this claim [4–8]. A large meta-analysis reviewing information used by intern selection committees to predict future performance of residents concluded that objective measures such as USMLE scores did this better than LORs and interview scores [6]. This seems to be at odds with results of the National Resident Matching Program survey of PDs who rate USMLE scores and LORs very highly when reviewing prospective interns [1].

What is evident after reviewing the literature is there is limited research reporting *how* LORs are used make decisions about applicants. While writing LORs is a time-honored tradition and it is hard to imagine the application without them, they take time to both read and write and are fraught with challenges [9]. The literature is replete with examples of how faculty struggle to communicate students’ performance in writing [10–14]. Themes that emerge from the literature reviewing LORs indicate that faculty use code language when writing letters lending themselves to being misconstrued [13, 14]. It could be speculated that LORs, much like narrative comments in evaluations and the Medical Student Performance Evaluation (MSPE), might suffer from being too vague and open to unconscious bias as well [15–19].

To address this gap in the literature we sought to build on work that was previously published reporting how residency PDs in pediatrics, surgery, and internal medicine interpreted three components of LORs [13, 14]. We asked PDs to rate commonly used phrases (e.g., “I give my highest recommendation” versus “performed at expected level”), letter features (e.g., academic rank of letter writer and overall length of letter), and applicant abilities (such as professionalism and trustworthiness). A majority of PDs confessed they used code words when describing applicants who were below average, and our results did confirm that PDs could “read between the lines” of LORs.

Using this data we developed a LOR scoring rubric to assess LORs submitted to a pediatric residency program. The objectives of this study were to use the rubric to analyze (1) the consistency of LOR ratings across raters, and (2) LOR components that contributed to overall impression of the LOR and candidate. One aim was to develop a tool that could be used in faculty development to write a more informative LOR.

## Materials and methods

Six medical educators with leadership positions in the pediatric residency program, clerkship program, and intern selection committee developed a LOR scoring rubric based on results of previously published studies looking at how PDs interpret components of LORs [13, 14]. As a starting point, the initial rubric contained three sections that mirrored the original survey (14 commonly used phrases,13 letter features, and 10 applicant abilities). To assess the commonly used letter phrases we assigned point values of 2 to -2 depending on the perceived strength or weakness of the phrase. To assess letter features we asked letter evaluators to rate how well they conveyed their depth of interaction with the applicant on a 5-point Likert scale (1 = poor, 5 = excellent). To assess applicant abilities we asked letter evaluators to identify words used in the LOR to describe an applicant and rate how well the letter writer described it on a 3-point Likert scale (1 = did not describe, 2 = described, 3 = described well).

Before finalizing the rubric, we read additional LORs to mine for any additional phrases or applicant abilities that were not included in the original survey using LORs of applicants on our final rank list from our 2016–2017 application season. They were randomly selected by our program coordinator and all identifying information (name of applicant, gender, name of letter writer, institution of letter writer) was redacted. For each round of development each letter evaluator was sent the same 5 LORs to evaluate using the scoring rubric. Using an iterative process we discussed letter phrases, features, and applicant abilities that were identified in the LORs and they were added if consensus was achieved. We reviewed 30 LORs for a total of six iterations until no further feedback was generated and theme saturation was achieved. The final rubric contained 21 letter phrases, 4 letter features, and 14 applicant abilities with 34 synonyms. There were two additional questions asking letter evaluators to rate (1) the overall quality of the LOR and (2) the overall impression of the quality of the applicant after reading the LOR on a 5-point Likert scale (1 = poor, 5 = excellent). A final copy of the rubric is available S1 File.

Using the finalized scoring rubric we graded 30 new and randomly selected LORs submitted to a moderate-sized pediatric residency program from our final rank list of 265 students. The LORs were evenly distributed based on final rank by our program with 10 top tertile, 10 middle tertile, and 10 lowest tertile. Inter-rater reliability was calculated with intraclass correlation coefficients (ICC(2,1)). Pearson (r) correlations and stepwise multivariate linear regression modeling predicted the overall quality of the letter (LORQ) and impression of the quality of the applicant (IC) after reading the LOR. The mean scores of letter phrases, features, applicant abilities, and position on final rank list were analyzed with ANOVA and the Bonferroni correction for multiple comparisons. A waiver of informed consent was approved and this study was approved by the Institutional Review Board of the Medical College of Wisconsin.

## Results and discussion

There was strong inter-rater reliability between letter evaluators for LOR commonly used phrases (ICC(2,1) = 0.82, P < 0.001)) and features (ICC(2,1) = 0.60, P <0.001)), but not applicant abilities (ICC(2,1) = 0.28, P < 0.001)). LORQ and IC scores were strongly correlated with commonly used phrases, letter features, and applicant abilities (r = 0.7–0.9, P < 0.001). For letter features, LORQ (R^2^ = 0.75, P < 0.001) was best predicted by writing about candidates’ specific abilities (b = 0.5), including a summative statement on the strength of the recommendation (0.3), and describing the depth of interaction with the applicant (0.2). The overall IC (R^2^ = 0.75, P < 0.001) was best predicted by the same 3 features as depicted in Table 1.

**Table 1:** Linear regression modelling of letter features predicting LORQ and IC.

Letter Feature	Letter Quality (LORQ)		Impression of Applicant (IC)	
	B	Sig (p)	B	Sig (p)
Description of applicant’s abilities	0.5	< .001	0.5	< .001
Summative statement on strength of recommendation	0.3	< .001	0.3	< .001
Description of depth of interaction with applicant	0.2	< .001	0.1	.020

For applicant abilities, LORQ (R^2^ = 0.47, P < 0.001) was best predicted by clinical reasoning (beta = 0.5), leadership (0.3), and communication skills (0.2). Trustworthiness, maturity, enthusiastic, team player, professionalism, compassionate, resilience, resourcefulness, inquisitiveness, and efficient did not factor into the model. The overall IC (R^2^ = 0.51, P < 0.001) was best predicted by the same 3 abilities. Team player, professionalism, compassionate, resilience, resourcefulness, inquisitiveness, and efficient did not factor into the model as shown in Table 2. Four or more applicant abilities that were rated as “described well” correlated with stronger LORQ and IC (r = 0.5–0.6, P < 0.001).

**Table 2:** Linear regression modelling of applicant abilities predicting LORQ and IC.

Applicant Ability	Letter Quality (LORQ)		Impression of Applicant (IC)	
	B	Sig (p)	B	Sig (p)
**Clinical reasoning**	0.4	< .001	0.4	< .001
**Leadership**	0.3	< .001	0.2	< .001
**Communication skills**	0.2	< .001	0.1	.018
**Work ethic**	0.2	.006	0.1	.021
**Trustworthiness**	**Did not factor into the model.**		0.1	.036
**Maturity**			-0.1	.035
**Enthusiastic**			-0.1	.004
**Team player**			**Did not factor into the model**	
**Professionalism**				
**Compassionate**				
**Resilience**				
**Resourcefulness**				
**Inquisitiveness**				
**Efficient**				

There were significant differences in mean scores between letter tiers for commonly used phrases, features and position on the final rank list (p<0.05) as shown in Table 3.

**Table 3:** Means and range scores for letter phrases, features and applicant abilities by rank tertile.

Letter Tier	Points for Commonly Used Phrases Per LOR Mean (range)	Combined Likert Rating Per LOR for Letter Features (maximum of 20 points)	Number of Applicant Abilities Described Per LOR	Position on Final Rank List
		Mean (range)	Mean (range)	Mean (range)
Top (n = 10)	5.7 (5.1–6.8)	16 (14–18)	7.6 (4.9–9.6)	41 (7–77)
Middle (n = 10)	2.9 (2.1–3.8)	15 (13–16)	7.1 (4.5–8.7)	137 (118–164)
Lowest (n = 10)	2.3 (2–2.5)	12 (11–14)	6.9 (4.4–7.9)	201 (169–234)
Sig (p)	.004*	.045*	.664	.001*

In this study looking at components of LORs that contributed to overall impression of the letter quality and impression of the applicant, we developed a scoring rubric that demonstrated good inter-rater reliability. Top tier LORs contained significantly more positive phrases, described the applicant better, and achieved a higher position on the final rank list than middle and lowest tier LORs. Select letter features and applicant abilities best predicted the strongest LORs and most favorable impression of the applicant. These results may help letter writers craft more informative LORs.

Developing a tool to reliably rate LORs was an important goal of this study. Prior research has cautioned that an objective system to evaluate LORs might prove too challenging to develop given the unique characteristics of the letter writer, applicant, and written language [20]. We were able to develop a rating tool that showed good consistency between raters and identified hallmarks of strong LORs that conveyed favorable impressions of applicants. While the standardized letter of recommendation has been suggested to eliminate the traditional LOR, it is hard to imagine reviewing the application without a richer narrative about applicants [9, 21–23].

Our results also shed some light into what pediatric residency program leadership may value in terms of applicant abilities when making selections about who to interview and rank in their programs. In the LORs reviewed for this study, clinical reasoning, leadership, and communication skills emerged as the top predictors of best letter quality and impression of the applicant. It is interesting that many other desirable abilities did not factor into the model, such as professionalism, when results of the 2020 National Resident Matching Program (NRMP) survey indicate that PDs in all specialties value professionalism as an important predictor of resident success in their program [24]. There was weak inter-rater reliability in our study for applicant abilities, which makes sense when one considers the abilities included in our rubric and the NRMP survey are inherently positive. It is the level of detail of the description of the applicant and their attributes in a LOR that we found most impactful to readers, and is the most important takeaway from our results.

Our results may best be suited for applications in faculty development as a majority of faculty report they receive little training for this important part of their job [25]. Because of this letter-writing can be a time-consuming task, especially when authors are asked to write multiple LORs per application season. To compose a top tier LOR our results suggest letter writers include a combination of the most positive phrases and describe both the depth of their interaction with the applicant and a number of their abilities with supporting details and a rich narrative. The rubric could also be used by faculty to evaluate LORs they write prior to uploading them into the Electronic Residency Application Service. A system that protects against a poorly written LOR (that scores few points on the rubric) on behalf of a strong applicant should exist as we know the quality of the LORs themselves contribute to high-stakes decisions and sway readers’ impressions of the applicant [13, 14]. Next steps for this rubric would be to standardize scoring criteria for faculty writing LORs. It is easy to write about the superstars, but applicants who are still developing deserve to be described well too. A rubric such as this may help faculty do that and would be important. PDs rate LORs second in importance only to USMLE Step 1 when selecting applicants to interview. With USMLE moving to pass/fail in 2022 LORs may become increasingly important [24].

This study was conducted at a single pediatric residency program, so may not be generalizable to other programs and specialties. Because the initial rubric was developed using the results of a survey of program PDs in pediatrics, surgery and internal medicine we know that PDs in all three specialties rated commonly used phrases, letter features, and applicant abilities very similarly so could speculate they would value a very similar LOR [13, 14]. Areas of divergence using this rubric might lie in the applicant abilities that are valued by the different specialties. Surgery PDs may value reading about an applicant’s technical abilities, and this is not an ability that is routinely used to describe an applicant for a residency in pediatrics. We must acknowledge our process for developing the rubric and ensuring all unique phrases and applicant abilities were included cannot be guaranteed given the unique nature of each LOR and the breadth of the English language. Further study is still needed to understand whether LORs predict performance in residency.

## Conclusions

The scoring rubric was consistent across raters and correlates with raters’ overall impression of the letter quality and impression of the applicant. Our results show promise for faculty development in quality letter writing.

## Supporting information

S1 File LOR scoring rubric. (PDF)
