# Supplementary material for: A novel faculty development tool for writing a letter of recommendation
Source: PLoS One. 2020 Dec 16;15(12):e0244016. doi: 10.1371/journal.pone.0244016 (PMC7743943; doi:10.1371/journal.pone.0244016)
Supplement: S1 File — (PDF) [file pone.0244016.s001.pdf]

Letter of Recommendation Scoring Rubric

1. Please circle any of the phrases that are written in the letter of recommendation you are reviewing:

Would like the applicant to stay at our institution	☐	2 points
Will be an asset to any program	☐	2 points
Exceeded expectations	☐	2 points
I give my highest recommendation	☐	2 points
Functioned at the level of an intern/resident	☐	2 points
I would rate them in the top (1%-5%) of students I have worked with	☐	2 points
They will be an outstanding resident/were an outstanding student/one of the best students I have worked with	☐	2 points
I recommend without reservation	☐	1 points
I highly recommend	☐	1 points
Functioned at the level of a fourth-year student (if a 3rd year student)	☐	1 points
I would rate them in the top (6-25%)	☐	1 points
They will be an excellent resident/were an excellent student	☐	1 points
I would rate them in the top (26%-50%)	☐	0 point
They will be a good resident/were a good student	☐	0 point
Solid performance	☐	0 points
I recommend	☐	0 points
Overcame personal setbacks	☐	0 point
Performed at expected level	☐	-1 point
Showed improvement	☐	-1 point
They struggled with…(any competency)	☐	-2 point
They had difficulty with…(any competency)	☐	-2 point

2. For the items below please rate how well the letter writer described each of the letter features using the following scale (1 = poor, 2 = fair, 3 = good, 4 = very good, 5 = excellent). This section is meant to grade the letter, not the applicant. For example, a score of "5" could give a negative or positive impression of the applicant.

	1	2	3	4	5
Description of the depth of interaction with the applicant:	○	○	○	○	○
Description of the applicant's specific abilities/qualities:	○	○	○	○	○
Summative statement on the strength of recommendation:	○	○	○	○	○
Inclusion of personal details about the applicant:	○	○	○	○	○

3. Please rate how well the letter writer described any of the applicant abilities/qualities listed below using the following scale (1 = did not describe, 2 = described, 3 = described well). A score of "3" could give a negative or positive impression of the applicant.

	1	2	3
Work ethic (Hardworking, Dedicated, Motivated, Conscientious)	○	○	○
Trustworthiness (Patient ownership, Reliable, Responsible)	○	○	○
Team player (Collaborative, Good to work with, Helpful)	○	○	○
Professionalism (Integrity, Role-Model)	○	○	○
Compassionate (Kind, Empathetic, Caring, Comforting Bedside Manner)	○	○	○

Maturity (Life experience)	◯	◯	◯
Resilience (Flexible, Persevered)	◯	◯	◯
Leadership (Poised, Innovative, Took Initiative)	◯	◯	◯
Resourcefulness (Quick learner, Self-directed)	◯	◯	◯
Inquisitiveness (Curious, Asked Good Questions)	◯	◯	◯
Communication Skills (Interacts well with others, Engaging, Sincere, Good listener, Organized)	◯	◯	◯
Clinical Reasoning Skills (Good judgment, Good differentials, Good knowledge)	◯	◯	◯
Efficient	◯	◯	◯
Enthusiastic	◯	◯	◯

4. Were there any additional words or phrases in the letter that you interpreted as describing the following abilities/qualities from section 3. Please enter them below:

Work ethic (Hardworking, Dedicated, Motivated, Conscientious)	
Trustworthiness (Patient ownership, Reliable, Responsible)	
Team player (Collaborative, Good to work with, Helpful)	
Professionalism (Integrity, Role-Model)	
Compassionate (Kind, Empathetic, Caring, Comforting Bedside Manner)	
Maturity (Life experience)	

Resilience (Flexible, Persevered)	
Leadership (Poised, Innovative, Took Initiative)	
Resourcefulness (Quick learner, Self-directed)	
Inquisitiveness (Curious, Asked Good Questions)	
Communication Skills (Interacts well with others, Engaging, Sincere, Good listener, Organized)	
Clinical Reasoning Skills (Good judgment, Good differentials, Good knowledge)	
Efficient	
Enthusiastic	
Other Abilities/Qualities not captured above	

For the below questions use the Likert scale 1 = poor, 2 = fair, 3 = good, 4 = very good, 5 = excellent

5. Please rate the overall quality of the letter of recommendation:

1 ○ 2 ○ 3 ○ 4 ○ 5 ○

6. Please rate your overall impression of the quality of the applicant after reading the letter of recommendation:

1 ○ 2 ○ 3 ○ 4 ○ 5 ○
